# Point-of-care Testing HbA1c screening for type 2 diabetes in urban and rural areas of China: a cost-effectiveness analysis

**DOI:** 10.3389/fpubh.2024.1438945

**Published:** 2024-07-30

**Authors:** Qing Shao, Xinglei Xie, Liu Wang, Lanyu Gao, Yuchen Hu, Yuwei Zhang

**Affiliations:** ^1^Department of Endocrinology and Metabolism, West China Hospital, Sichuan University, Chengdu, China; ^2^Center for Diabetes and Metabolism Research, West China Hospital of Sichuan University, Chengdu, China

**Keywords:** type 2 diabetes, Point-of-care Testing, glycated hemoglobin assay, capillary glucose, cost-effectiveness analysis, Markov model

## Abstract

**Background:**

Point-of-care Testing (POCT) glycosylated hemoglobin (HbA1c) is a convenient, cheap, effective and accessible screening method for type 2 diabetes in rural areas and community settings that is widely used in the European region and Japan, but not yet widespread in China. The study is the first to evaluate the cost-effectiveness of POCT HbA1c, fasting capillary glucose (FCG), and venous blood HbA1c to screen for type 2 diabetes in urban and rural areas of China, and to identify the best socio-economically beneficial screening strategy.

**Methods:**

Based on urban and rural areas in China, economic models for type 2 diabetes screening were constructed from a social perspective. The subjects of this study were adults aged 18–80 years with undiagnosed type 2 diabetes. Three screening strategies were established for venous blood HbA1c, FCG and POCT HbA1c, and cost-effectiveness analysis was performed by Markov models. One-way sensitivity analysis and probabilistic sensitivity analysis were performed on all parameters of the model to verify the stability of the results.

**Results:**

Compared with FCG, POCT HbA1c was cost-effective with an incremental cost-utility ratio (ICUR) of $500.06/quality-adjusted life year (QALY) in urban areas and an ICUR of $185.10/QALY in rural areas, within the willingness-to-pay threshold (WTP = $37,653). POCT HbA1c was cost-effective with lower cost and higher utility compared with venous blood HbA1c in both urban and rural areas. In the comparison of venous blood HbA1c and FCG, venous blood HbA1c was cost-effective (ICUR = $20,833/QALY) in urban areas but not in rural areas (ICUR = $41,858/QALY). Sensitivity analyses showed that the results of the study were stable and credible.

**Conclusions:**

POCT HbA1c was cost-effective for type 2 diabetes screening in both urban and rural areas of China, which could be considered for future clinical practice in China. Factors such as geographic location, local financial situation and resident compliance needed to be considered when making the choice of venous blood HbA1c or FCG.

## 1 Introduction

Type 2 diabetes poses one of the biggest public health problems affecting hundreds of millions of people worldwide, with significant morbidity and mortality implications for individual patients and families as well as considerable economic impact on national health care systems (1). Type 2 diabetes is associated with both macrovascular and microvascular complications, which greatly increase both the disability and mortality rates. It is estimated that about 6 million people with type 2 diabetes in China suffer from more than one complication (2). Among urban Chinese residents, 73–81% of direct diabetes-related medical costs are spent on the treatment of diabetic complications (3).

A meta-analysis showed an increased risk of all-cause mortality and cardiovascular mortality in patients with type 2 diabetes associated with blood glucose levels (4). The United kingdom Prospective Diabetes Study results showed that a mean glycosylated hemoglobin (HbA1c) of 53 mmol/mol (7.0%) reduced the risk of diabetes-related endpoints by 12–32% compared to a mean HbA1c of 63 mmol/mol (7.9%) (5). The prevalence and mortality of type 2 diabetes in rural areas of China are increasing yearly (6, 7). However, the frequency of self-monitoring of blood glucose in Chinese rural residents is relatively low, and the glycemic target attainment rate is < 19% (8). A study from Taiwan (9) reported that patients with type 2 diabetes in rural areas had a higher risk of developing chronic complications than those in urban areas. These studies all demonstrate the urgency of early diagnosis of type 2 diabetes and the importance of early glycemic target attainment to prevent further complications.

The oral glucose tolerance test (OGTT) is the gold standard for diagnosing type 2 diabetes. HbA1c is a commonly used indicator for monitoring glycemic control in clinical practice, as well as one of the diagnostic criteria for diabetes (10). However, the strict quality control requirements for venous HbA1c render it unavailable in many remote areas of rural China. Fingertip fasting capillary glucose (FCG) is the most common method for initial screening of diabetes and self-monitoring of blood glucose in the daily life of Chinese residents. However, FCG is affected by short term lifestyle changes, as well as environmental humidity and temperature, with poor repeatability and stability, and the sensitivity of screening for type 2 diabetes in the Chinese population is only 65.1% (11). Point-of-care Testing (POCT) glycosylated hemoglobin (HbA1c) is a minimally invasive HbA1c test that provides rapid results by collecting the subject's fingertip blood and analyzing it immediately at the sampling site. Importantly, POCT HbA1c analyzers are currently used in about 75% of European healthcare facilities (12). In Japan, a community-based specimen measurement office was initiated and offered POCT HbA1c testing in 2014, and a cost-effectiveness analysis in 2018 showed that POCT HbA1c was more effective and less costly compared with the status quo (HbA1c testing that was available only during Specific Health Checkup visits and conventional opportunistic screening in clinics) (13). Studies in recent years have shown that most POCT HbA1c measurement instruments can satisfy the quality specifications with high accuracy and sensitivity (12, 14, 15). Nevertheless, in China, POCT HbA1c testing is not yet widely used in clinical practice and relatively few clinical studies have been conducted. There are no cost-effectiveness studies of POCT HbA1c in China.

In this study, a cost-effectiveness analysis of three screening strategies for type 2 diabetes (POCT HbA1c, venous blood HbA1c and FCG) was conducted in rural and urban areas from a social perspective to identify the most economically efficient screening method. This is the first cost-effectiveness study of POCT HbA1c in China, which could provide a foundation and reference for the application of POCT HbA1c in type 2 diabetes screening in urban and rural areas of China.

## 2 Methods

### 2.1 Study design

This study presented a social perspective to construct diabetes screening strategies based on venous HbA1c, FCG, and POCT HbA1c by decision tree model, and performed cost-effectiveness analysis of the three screening methods by Markov model to determine the economically efficient strategy in urban and rural areas of China, respectively. Treeage Pro 2022 software was used for model construction and computation. We searched the databases of China National Knowledge Infrastructure, PubMed, and Web of Science, selected representative large retrospective studies, cohort studies, and relevant cost-effectiveness analysis studies from 2012 to 2022, and referred to annual government statistical reports, local government reports and price lists to obtain the parameters required for decision tree model and Markov models.

The major literature inclusion criteria were as follows: (1) Subjects: Adults (≥18 years old) without a diagnosis of type 2 diabetes, Chinese population was preferred, if there was no relevant study in the Chinese population, Asian population (e.g., Japan) was preferred, followed by European population. (2) Study types: (1) Transition probabilities: large cohort studies or cost-effectiveness analysis studies, of which cohort studies had a follow-up period of not < 3 years; (2) Costs: large cross-sectional studies, retrospective studies, or cost-effectiveness analysis studies in Chinese regions; (3) Utilities: cost-effectiveness analysis studies (preferred in Chinese regions). (3) Literature publication period: 2012–2022. Exclusion criteria included: (1) Study participants were children, adolescents, or pregnant women, with a previous diagnosis of pre-diabetes or type 1 diabetes or a specific type of diabetes. (2) Studies not formally published, such as conference abstracts. (3) Written and published in languages other than Chinese and English.

The Markov chain Monte Carlo simulation method was used to estimate the disease process of 10 million adults (18 years and older) with no previous diagnosis of type 2 diabetes (exclusion of prediabetes, type 1 diabetes, or specific types of diabetes). The age and the male-to-female sex ratio of the baseline population was calculated based on the China Statistical Yearbook 2021 (16) (Supplementary Table 1). In this study, we used 1 year as a circulation period, and the model termination condition is age ≥80 years. Due to the long and continuous disease course of type 2 diabetes, the discretization process of the Markov model may produce some error, so the model was corrected by half-cycle correction. In addition, this study made assumptions about the model: (1) all type 2 diabetes patients were in a stable baseline state (without complications) before entering the long-term Markov model. (2) Transitions between states in the Markov model of the subject do not affect the next cycle of transitions (17).

### 2.2 Model construction and setup

This study included decision tree models for three screening strategies and Markov models for simulating disease progression.

**Figure 1:**
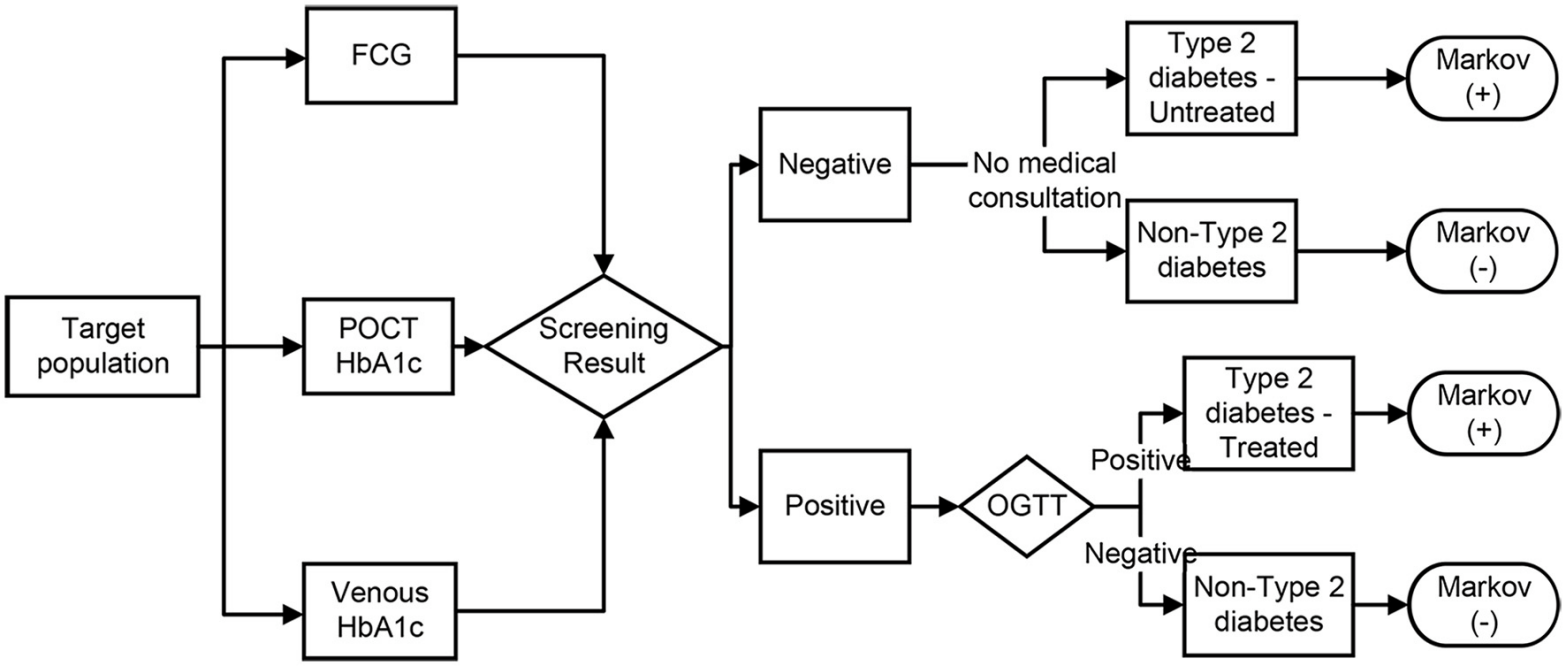
Decision model for the three screening strategies. FCG, fasting capillary blood glucose; POCT, point-of-care test; OGTT, oral glucose tolerance test. Screening positive: POCT HbA1c test value ≥43.6 mmol/mol (6.14%) (15), venous blood HbA1c test value ≥44.8 mmol/mol (6.25%) (18), FCG test value ≥6.1 mmol/L (11), OGTT test with fasting plasma glucose ≥7.0 mmol/L and/or 2 h plasma glucose after oral 75 g of glucose powder ≥11.1 mmol/L (10).

The decision model in this study simulated the process of screening for type 2 diabetes in the population using POCT HbA1c, FCG, and venous blood HbA1c (as shown in Figure 1). Following the screening test, positive individuals would be further tested with an OGTT. If OGTT is positive, type 2 diabetes would be diagnosed and treatment would be initiated [enter the Markov (+) model], and if OGTT is negative, misdiagnosis would be considered [enter the Markov (-) model]. Those who screen negative would not be further examined and treated [Markov (-) model], but if the subject are patients with type 2 diabetes, they are considered to be missed [Markov (+) model]. The sensitivity and specificity of the different screening methods determine the rate of missed and misdiagnosis of screening, and influence the disease progression process of the subject.

**Figure 2:**
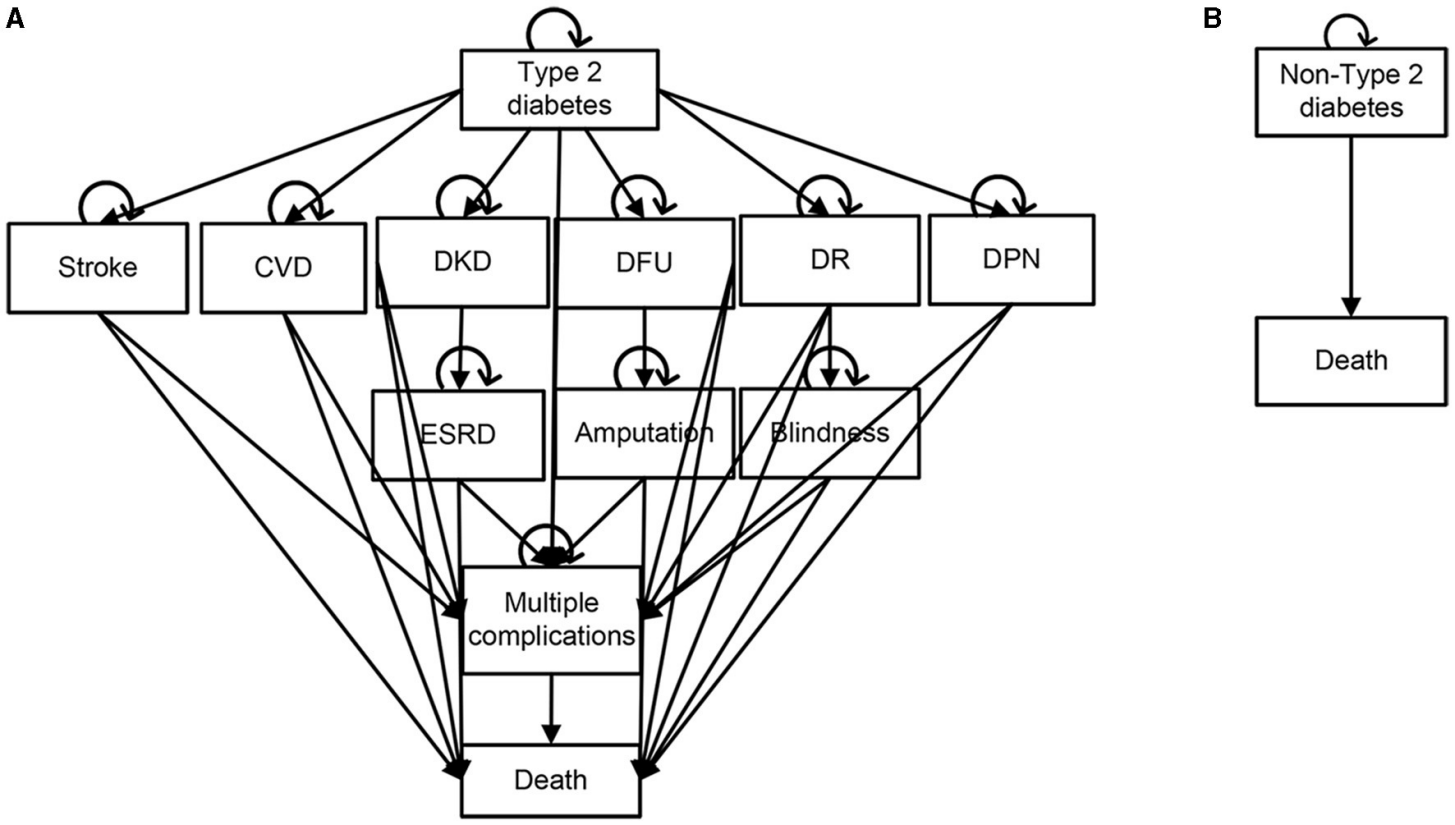
Schematic diagrams of Markov model. **(A)** Type 2 diabetes [Markov(+)] model. **(B)** Non-type 2 diabetes [Markov(-)] model. CVD, Cardiovascular Disease; ESRD, End-Stage Renal Disease; DKD, Diabetic kidney disease; DFU, Diabetic foot ulcer; DR, Diabetic retinopathy; DPN, Diabetic peripheral neuropathy.

Markov (+) model (Figure 2A): including type 2 diabetes without complications, type 2 diabetes with various related complications and death. The complications included macrovascular complications (cardiovascular disease, stroke) and microvascular complications (diabetic kidney disease, diabetic foot ulcers, diabetic retinopathy, and diabetic peripheral neuropathy). In particular, diabetic kidney disease, diabetic foot ulcers and diabetic retinopathy could progress to end-stage renal disease, amputation and blindness. Type 2 diabetes with or without complications may cause multiple complications or death. In the process, different complications affected the health status of patients with type 2 diabetes in different ways, resulting in corresponding costs.

Markov (-) model (Figure 2B): including non-type 2 diabetes and death. Subjects in this model may continue to maintain the non-type 2 diabetes state or death.

### 2.3 Costs

All costs of the three screening methods for type 2 diabetes were collected from the social perspective, including direct medical costs, direct non-medical costs and indirect costs. The direct medical costs were the medical resources consumed by the disease, such as registration fees, test fees and medical staff time costs. Registration and testing fees were referenced to local health service prices, and medical staff time costs are obtained from the average salary of medical staff published by the National Bureau of Statistics of China in 2021 (19). Direct non-medical costs were costs incurred in seeking and waiting for test results (e.g., transportation, accommodation, etc.). The direct non-medical costs in urban areas were obtained from a cost-effectiveness study in Chinese communities (20), and direct non-medical costs in rural areas were obtained from a cross-sectional study in rural southwest China (21). Indirect costs were costs incurred by subjects for time spent on medical visits and waiting, calculated as the average hourly wage of the Chinese population (22). The detailed cost components and model input parameters were shown in the Supplementary Table 2 and Supplementary Table 3A.

After the development of complications in type 2 diabetes, it is difficult to calculate the direct non-medical costs and indirect costs resulting from different types of complications due to the large differences in disease duration, treatment options and prognosis, so only the direct medical costs of type 2 diabetes complications were included in this study as approximate total costs. Costs of complications were derived from previously published cross-sectional studies based on Chinese populations, retrospective studies, and relevant cost-effectiveness studies (as shown in Supplementary Table 3B). All costs were converted to US dollars at an exchange rate of US$1.0 = CNY6.4 (2022). Because the study period was >1 year and there may be socioeconomic inflation, this study discounted the future costs and health outputs from the screening measures (the discount rate was taken as 5%). A sensitivity analysis was also performed for discount rates between 0 and 8%.

### 2.4 Utilities

The utility values for this study were set to quality-adjusted life years (QALYs). The utility values were derived from cost-effectiveness studies based on Chinese populations, except for the utility values for diabetic neuropathy and multiple complications, which were referenced from cost-utility analysis studies in European regions. The specific parameter values were shown in Supplementary Table 4.

### 2.5 Transition probabilities

The prevalence of type 2 diabetes in urban and rural areas in this study was derived from a Chinese cross-sectional study in 2020 (23). The sensitivity and specificity of the three screening strategies were derived from diagnostic studies based on Chinese populations (11, 15, 18). The transition probabilities of various complications were mainly derived from cohort studies and cost effectiveness analyses based on Chinese populations, some of which were referred to foreign studies because no relevant studies were available in China. In addition, a 10-year cohort study showed that in patients with HbA1c of 7–8%, the risk ratio (HR) for microvascular complications was 1.391, HR for macrovascular complications was 1.287, and HR for death was 1.290 (24). Therefore, in this study, for patients with missed diagnosis type 2 diabetes, the transition probabilities of complications were set as the original transition probabilities multiplied by the corresponding HR.

In terms of urban-rural differences, a study in Taiwan, China, showed that the risk of cardiovascular events in type 2 diabetes was 1.15 times higher in rural areas than in urban areas, and the HRs for stroke, blindness, lower limb ulcers, and end-stage renal disease were 1.25, 2.09, 1.42, and 1.15, respectively (9). Therefore, in this study, the transition probabilities of complications in rural areas were set as the original transition probabilities multiplied by the corresponding HR.

The specific transition probability parameter values and references were detailed in Supplementary Table 5. All transition probabilities were subjected to sensitivity analysis.

### 2.6 Evaluation methods

This study conducted a cost-effectiveness analysis and used the incremental cost-utility ratio (ICUR) as an outcome indicator to explain the results of the cost-effectiveness analysis. The ICUR was the cost per additional QALY for the subject, which was compared to the willingness-to-pay threshold (WTP) to assess the economic advantages of different screening strategies. The WTP is usually 1–3 times the gross domestic product (GDP) per capita. The GDP per capita in China in 2021 is ~$12,551 (25), and the WTP of this study was taken as 3 times the GDP per capita ($37,653).

### 2.7 Sensitivity analysis

To evaluate the extent to which factors such as model building, parameter differences and evaluation methods affected the study results during the study, we performed one-way sensitivity analysis and probabilistic sensitivity analysis on all input model parameters (including costs, prevalence of type 2 diabetes, screening effectiveness, transition probabilities, utilities, and discount rate) to verify whether the study results were stable and reliable. In the one-way sensitivity analysis, the ranges of different parameters were varied using the 95% confidence intervals given in the references, and if only the mean value of the parameter was reported in the data source, the base data ± 20% was used as the range of variation of the parameter for the sensitivity analysis. The probabilistic sensitivity analysis was designed to assess the combined effect of multiple parameter changes on the model results. All parameters were set to probability distributions for 1,000 iterations.

(1) For dichotomous variables, if the number of occurrences and the sample size were reported in the previous literature, a beta distribution is appropriate and the probability density function is as follows. Where α represents the number of events that occurred and β represents the number of events that did not occur. The parameters for fitting the beta distribution in this study are shown in Supplementary Table 6A.

$$\begin{array}{l} f_{\left(x;\alpha ;\beta \right)}=\frac{\Gamma \left(\alpha +\beta \right)}{\Gamma \left(\alpha \right)\Gamma \left(\beta \right)}x^{\alpha -1}{\left(1-x\right)}^{\beta -1} \end{array}$$

(2) Health costs or prices are usually distributed as skewed. This study assumes that both screening and complication costs follow a lognormal distribution, taking the log mean as the median and the standard deviation as 10% of the mean. The probability density function is as follows (μ denotes the log mean and σ denotes the log standard deviation). The parameters for fitting the lognormal distribution in this study are shown in Supplementary Table 6B.

$$\begin{array}{l} f\left(x\right)=\frac{1}{x\sigma \sqrt{2\pi }}e^{\left(\frac{-{\left(\ln\left(x\right)-\mu \right)}^{2}}{2\sigma^{2}}\right)} \end{array}$$

(3) All other variables were fit to a uniform distribution as the parameters of the particular distribution could not be derived from previous literature or from the actual situation. The parameters for the uniform distribution utilized in this study are presented in Supplementary Table 6C.

## 3 Results

### 3.1 Cost-effectiveness analysis results

The cost-effectiveness analysis results were shown in Table 1.

**Table 1:** Results of cost-effectiveness analysis of three screening strategies.

	**Cost ($)**	**Incremental cost ($)**	Utility (QALY)^*^	**Incremental utility (QALY)**	**C/U**	**ICUR ($/QALY)**
**Urban area**						
FCG	1,911.10	-	27.12	-	70.46	-
POCT HbA_1c_	1,956.44	45.33	27.21	0.09	71.90	500.06
Venous blood HbA_1c_^†^	2,536.09	579.65	27.15	−0.06	93.41	−10,028.50
Venous blood HbA_1c_^‡^	2,536.09	624.99	27.15	0.03	93.41	20,833
**Rural area**						
FCG	2,051.15	-	26.54	-	77.29	-
POCT HbA_1c_	2,062.00	10.85	26.60	0.06	77.52	185.10
Venous blood HbA_1c_^†^	2,888.31	826.31	26.56	−0.04	108.75	−22,105.23
Venous blood HbA_1c_^‡^	2,888.31	837.16	26.56	0.02	108.75	41,858

^*^Utility: Quality-adjusted life year (QALY).

^†^Venous blood HbA_1c_ compared with POCT HbA_1c_.

^‡^Venous blood HbA_1c_ compared with FCG.

ICUR, incremental cost-utility ratio; HbA_1c_, glycated hemoglobin; FCG, fasting capillary blood glucose; POCT, point-of-care test.

In urban areas, the lifetime costs per capita for FCG, POCT HbA1c, and venous blood HbA1c were $1,911.10, $1,956.44, and $2,536.09, respectively. In terms of utility, POCT HbA1c had the highest QALY (QALY = 27.21), which was 0.09 and 0.06 higher than FCG (QALY = 27.12) and venous blood HbA1c (QALY = 27.15), respectively. Compared with FCG, the ICUR for POCT HbA1c was $500.06/QALY, which was within the WTP ($37,653) threshold. Compared with venous blood HbA1c, POCT HbA1c had lower cost and higher utility with cost-effectiveness. Venous blood HbA1c was cost-effective with an ICUR of $20,833/QALY compared to FCG, within the WTP threshold.

In rural areas, the lifetime costs per capita for FCG, POCT HbA1c, and venous blood HbA1c were $2,051.15, $2,062.00, and $2,888.31, respectively. In terms of utility, POCT HbA1c had the highest QALY (QALY = 26.60), which was 0.06 and 0.04 higher than FCG (QALY = 26.54) and venous blood HbA1c (QALY = 26.56), respectively. Compared with FCG, the ICUR of POCT HbA1c was $185.10/QALY, which was within the WTP ($37,653) threshold. Compared with venous blood HbA1c, POCT HbA1c had lower cost and higher utility with cost-effectiveness. Venous blood HbA1c had an ICUR of $41,858/QALY compared to FCG, which is greater than the WTP threshold.

**Figure 3:**
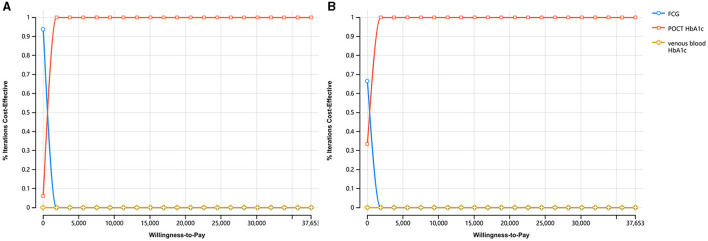
The cost-utility acceptability curve of the three screening strategies. **(A)** The cost-utility acceptability curve in urban areas. **(B)** The cost-utility acceptability curve in rural areas.

The cost-utility acceptability curve of the three screening strategies in both urban and rural areas were shown in Figure 3. The probability that POCT HbA1c was cost-effective was 100% for each when the WTP = $37,653.

### 3.2 Sensitivity analysis results

Due to the large number of input parameters to the models, this study presented the results of the one-way sensitivity analyses of all the parameters together through the tornado diagrams of ICUR values (Supplementary Figure 1). In the tornado diagrams, a horizontal band was generated for each analyzed variable, the length of which represented the magnitude of its impact on the model results, and all parameters were ordered from top to bottom according to the magnitude of their impact on the models. The vertical lines in the figure represented the mean ICUR values obtained from the model operations. In comparison of POCT HbA1c with FCG and venous blood HbA1c, the ICUR values for each parameter did not exceed the WTP of this study when the parameters were varied within their sensitivity analysis in both urban and rural areas, and the results of the study were relatively stable. In comparison of venous blood HbA1c with FCG, the ICUR value for each parameter did not exceed the WTP in urban areas, while in rural areas, the factors that had a greater impact on the results were the discount rate, prevalence of type 2 diabetes in rural areas, transition probability of multiple complications, etc. the ICUR value exceeded the WTP, which had an impact on the study results considering the variation of the above parameters within the reference range.

**Figure 4:**
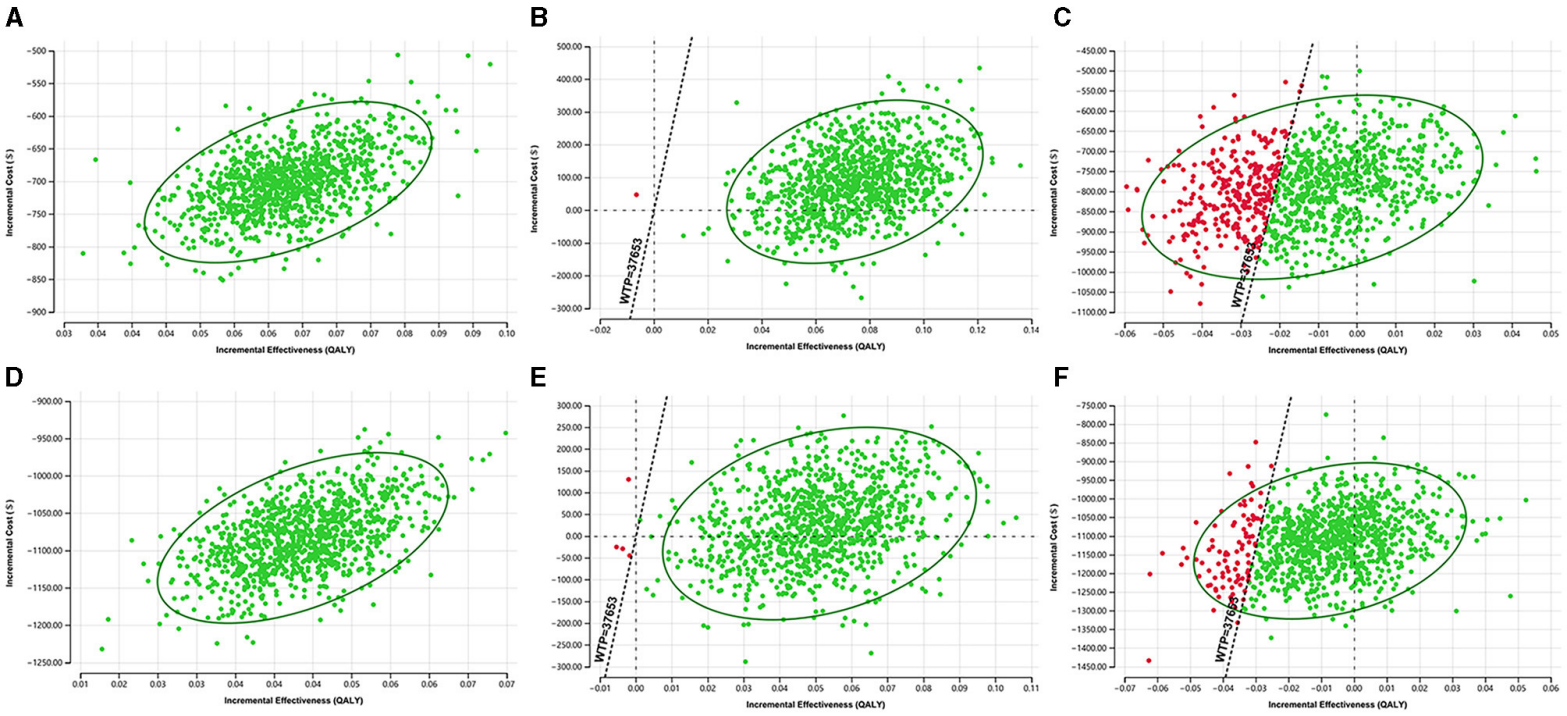
The probabilistic sensitivity analysis results of the ICUR in three screening strategies. **(A)** POCT HbA1c vs. FCG in urban areas. **(B)** POCT HbA1c vs. venous blood HbA1c in urban areas. **(C)** Venous blood HbA1c vs. FCG in urban areas. **(D)** POCT HbA1c vs. FCG in rural areas. **(E)** POCT HbA1c vs. venous blood HbA1c in rural areas. **(F)** Venous blood HbA1c vs. FCG in rural areas.

The scatter plots of ICUR for the three screening strategies in the probabilistic sensitivity analysis were shown in Figure 4. When WTP = $37,653, in urban areas, the probability that POCT HbA1c was cost-effective was 99.9% compared to FCG and 100% compared to venous blood HbA1c. The probability that venous blood HbA1c was cost-effective compared to FCG was 30%. In rural areas, the probability that POCT HbA1c was cost-effective was 99.6% compared to FCG and 100% compared to venous blood HbA1c. The probability that venous blood HbA1c was cost-effective compared with FCG was 9.1%.

## 4 Discussion

### 4.1 Analysis of the economic evaluation

In cost-effectiveness analysis, compared with FCG, the ICUR values of POCT HbA1c were within the WTP ($37,653) threshold in urban and rural models, and POCT HbA1c had lower cost and higher utility compared with venous blood HbA1c, suggesting that POCT HbA1c was cost-effective among the three screening strategies in both urban and rural areas of China. While the screening cost of POCT HbA1c was higher than that of FCG, its higher sensitivity in screening reduced the rate of missed diagnosis of type 2 diabetes, which further reduced the incidence and costs of type 2 diabetes-related complications. Compared with venous blood HbA1c, POCT HbA1c had lower cost and higher utility values in both urban and rural areas, which was related to the lower screening cost and higher screening sensitivity of POCT HbA1c.

In the comparison in venous blood HbA1c and FCG, the ICUR of venous blood HbA1c was $20,833/QALY (within the WTP threshold) in urban areas, while the ICUR was $41,858/QALY (greater than the WTP threshold) in rural areas. Venous blood HbA1c was superior to FCG in terms of sensitivity and specificity in screening for type 2 diabetes resulting in higher utility values. However, the cost of screening for venous blood HbA1c was high as well as the direct non-medical and indirect costs such as transportation, lost wages, and other costs associated with screening for venous blood HbA1c in rural areas. The results indicated that venous blood HbA1c was cost-effective in urban areas whereas FCG was more optimal in rural areas.

There are important urban-rural disparities when considering medical costs and calculating relative utilities, although the gap between the prevalence of diabetes in urban and rural areas is decreasing yearly (26, 27). However, medical services, medical equipment, and healthcare systems in rural areas of China still differ significantly from those in urban areas (28). The direct non-medical costs (e.g., transportation, accommodation, etc.) and indirect costs (e.g., lost wages, etc.) required by rural residents for disease diagnosis and treatment were higher, which was the principal explanation why the costs of the three screening strategies in rural areas were higher than those in urban areas in this study. Additionally, due to factors such as literacy and health education, rural residents were found to have less awareness of self-monitoring of blood glucose, poorer adherence to treatment, and a lower rate of attainment of glycemic target. Consequently, these factors resulted in a higher risk of diabetes-related complications, consistent with the lower health utility values in rural areas compared with urban areas in this study.

### 4.2 Sensitivity analysis confirms the stability of the study results

Because the parameters such as costs, transition probabilities and utility values required for this study were obtained from relevant cohort studies, cross-sectional studies, and cost-effectiveness analysis studies in China and abroad, one-way sensitivity analysis and probabilistic sensitivity analysis were performed for both urban and rural models to assess the effect of changes in input parameters on the study results. In the model of POCT HbA1c compared with FCG and venous blood HbA1c, the results of this study were stable when the parameters varied within the reference range or ±20%, either when a single parameter changed or when multiple parameters changed simultaneously.

In the model comparing venous blood HbA1c with FCG in rural areas, the one-way sensitivity analysis suggested that the ICUR value was greater than the WTP threshold and the results were unstable; the main influencing factors included the discount rate, prevalence of type 2 diabetes in rural areas and transition probability of multiple complications. The probabilistic sensitivity analysis showed that the probability of venous blood HbA1c had cost-effectiveness in urban areas was 30%, and the probability in rural areas was only 9.1%. Therefore, factors such as geographic location, local financial situation, and resident compliance needed to be considered when making the choice of venous blood HbA1c or FCG.

### 4.3 Current status in the application and quality control of POCT HbA1c

Currently, POCT HbA1c has being used for type 2 diabetes screening in Japan (13) and many European regions (12), whereas it is not widely used in China. This may be related to the lack of relevant Chinese studies on POCT HbA1c and the uncertainty of its accuracy and quality control. To this point, a meta-analysis found that POCT HbA1c results showed an overall negative bias and that the diagnostic cut-point values differed between regions and populations (29). With continued progress in the development of the POCT assay technology, small-sample Chinese studies have confirmed that POCT HbA1c showed good accuracy, precision and resistance to interference (30, 31). One example from 2017 reported on 842 community-based physical examinations that demonstrated POCT HbA1c testing diagnosed diabetes with a cut-point value of 6.14% and an AUC of 0.911, highly accurate and precise (15). However, compared with European countries, there are relatively few clinical studies on POCT HbA1c in China.

### 4.4 Strengths and limitations

The study is the first to conduct a cost-effectiveness analysis of POCT HbA1c in China. Three screening strategies were investigated: POCT HbA1c, FCG and venous blood HbA1c. They were constructed from a social perspective, and their long-term effects on the duration and economics of type 2 diabetes were evaluated in urban and rural areas of China. In the Markov model, we set up six major complications of type 2 diabetes, including microvascular complications (diabetic kidney disease, diabetic foot ulcers, diabetic retinopathy, and diabetic peripheral neuropathy) and macrovascular complications (stroke and cardiovascular disease), with assumptions about disease progression, including end-stage renal disease, amputation, and blindness. Also, we included an analysis of multiple complications, considering that many patients in the clinic are accompanied by more than one complication. Compared with previous studies of POCT HbA1c (13), this model was more comprehensive and a closer approximation to reality. The intent was to mimic real-life clinical practice and management in rural and community healthcare organizations in China.

There are limitations to our study. First, the models constructed in this study were relatively simplified economic models, despite including comprehensive assumptions about different complications and progression states of type 2 diabetes, different interventions following screening that could impact the development and course of the disease, morbidity and mortality of different complications, probabilities of interconversion or combination of complications, and costs in the real world. Second, because some model parameters such as the transition probability and cost of multiple complications have not been studied in China, we used data results from large prospective cohort studies derived from other countries. For the status of multiple complications, we set the same transition probability and cost due to limited study data. Third, because the utilities of type 2 diabetes and complications are related to subject compliance, the model set the compliance of the population at 100%, recognizing the actual compliance of the real-life population might be quite different. Finally, there were many brands of POCT HbA1c devices in the Chinese and international markets, and the diagnostic cut-point values and accuracies of different brands of devices are different, which could influence the study results.

## 5 Conclusion

In summary, this study suggested that POCT HbA1c was cost-effective for type 2 diabetes screening in both urban and rural areas of China, which could be considered for future clinical practice in China. Factors such as geographic location, local financial situation, and resident compliance needed to be considered when making the choice of venous blood HbA1c or FCG.

## Data availability statement

The raw data supporting the conclusions of this article will be made available by the authors, without undue reservation.

## Ethics statement

The studies involving humans were approved by Ethics Committees of West China Hospital. The studies were conducted in accordance with the local legislation and institutional requirements. Written informed consent for participation was not required from the participants or the participants' legal guardians/next of kin in accordance with the national legislation and institutional requirements.

## Author contributions

QS: Data curation, Formal analysis, Investigation, Methodology, Writing – original draft, Writing – review & editing. XX: Data curation, Formal analysis, Investigation, Methodology, Writing – original draft, Writing – review & editing. LW: Investigation, Methodology, Project administration, Supervision, Validation, Writing – review & editing. LG: Investigation, Methodology, Supervision, Validation, Writing – review & editing. YH: Investigation, Methodology, Supervision, Validation, Writing – review & editing. YZ: Conceptualization, Funding acquisition, Investigation, Methodology, Project administration, Resources, Supervision, Writing – review & editing.
