# Supplementary material for: Point-of-care Testing HbA1c screening for type 2 diabetes in urban and rural areas of China: a cost-effectiveness analysis
Source: Front Public Health. 2024 Jul 30;12:1438945. doi: 10.3389/fpubh.2024.1438945 (PMC11319179; doi:10.3389/fpubh.2024.1438945)
Supplement: Supplementary file 1 [file Data_Sheet_1.docx]

Supplementary Material

Supplementary Table S1. Age and gender characteristics of the baseline population

| Parameters | Values | Distribution |
| --- | --- | --- |
| Age(years) | 45.70±17.28 | normal distribution |
| Gender | Male 51.05%  Female 48.92% | bicategory |

Supplementary Table S2. Cost Composition of Three Strategies for Screening Type 2 Diabetes

| Cost Components | Urban | | | | Rural | | | |
| --- | --- | --- | --- | --- | --- | --- | --- | --- |
|  | OGTT | POCT HbA1c | FCG | Venous HbA1c | OGTT | POCT HbA1c | FCG | Venous HbA1c |
| Direct medical costs | 16.36 | 4.34 | 3.24 | 19.58 | 16.36 | 4.34 | 3.24 | 19.58 |
| registration fee | 6.88 | 0 | 0 | 6.88 | 6.88 | 0 | 0 | 6.88 |
| examination fee | 6.88 | 3.9 | 2.8 | 11.4 | 6.88 | 3.9 | 2.8 | 11.4 |
| medical staff time | 2.6 | 0.44 | 0.44 | 1.3 | 2.6 | 0.44 | 0.44 | 1.3 |
| Direct non-medical costs | 18.5 | 0 | 0 | 18.5 | 44.6 | 0 | 0 | 44.6 |
| Indirect costs | 21.9 | 0 | 0 | 21.9 | 21.9 | 0 | 0 | 21.9 |
| Total | 56.76 | 4.34 | 3.24 | 59.98 | 82.86 | 4.34 | 3.24 | 86.08 |

FCG: fasting capillary blood glucose. POCT: point-of-care test. OGTT: oral glucose tolerance test.

Supplementary Table S3A. Annual Cost of Three Strategies for Screening Type 2 Diabetes

|  | Cost | Urban | | Rural | |
| --- | --- | --- | --- | --- | --- |
|  |  | Values | Ranges | Values | Ranges |
| Non-diabetes/Type 2 diabetes-screen negative | Venous blood HbA1c | 59.98 | 47.98-71.98 | 86.08 | 68.86-103.30 |
|  | POCT HbA1c | 4.34 | 3.47-5.21 | 4.34 | 3.47-5.21 |
|  | FCG | 3.24 | 2.59-3.89 | 3.24 | 2.59-3.89 |
| Non-diabetes - screen positive | Venous blood HbA1c+OGTT | 113.3 | 90.64-135.96 | 165.5 | 132.3-198.6 |
|  | POCT HbA1c+OGTT | 61.1 | 48.88-73.32 | 87.2 | 69.76-104.64 |
|  | FCG+OGTT | 60 | 48-72 | 86.1 | 68.88-103.32 |
| Type 2 diabetes - screen positive | Type 2 diabetes+ Venous blood HbA1c+OGTT | 477.3 | 381.84-572.76 | 529.5 | 423.6-635.4 |
|  | Type 2 diabetes+POCT HbA1c+OGTT | 425.1 | 340.08-510.12 | 451.2 | 360.96-541.44 |
|  | Type 2 diabetes+FCG+OGTT | 424 | 339.2-508.8 | 450.1 | 360.08-540.12 |

FCG: fasting capillary blood glucose. POCT: point-of-care test. OGTT: oral glucose tolerance test.

Supplementary Table S3B. Annual Cost of Type 2 Diabetes and Complications

| Cost | Values | Ranges | References |
| --- | --- | --- | --- |
| Type 2 diabetes without complications | 364 | 291.2-436.8 | (1) |
| DKD | 1692 | 1253-2255 | (2) |
| ESRD | 14241 | 13571-15031 | (3) |
| DFU | 2334.7 | 1867.76-2801.64 | (4) |
| Amputation | 4307.4 | 3445.92-5168.88 | (5) |
| DR | 741.94 | 593.552-890.328 | (6) |
| Blindness | 3765 | 3012-4518 | (7) |
| CVD | 1222.66 | 978.13-1467.19 | (8) |
| Multiple complications | 2994 | 2395.2-3592.8 | (2) |
| DPN | 1102 | 691-1628 | (2) |
| Stroke | 1662.03 | 1630.47-1693.75 | (9) |

CVD : Cardiovascular Disease. ESRD: End-Stage Renal Disease. DKD: Diabetic kidney disease. DFU: Diabetic foot ulcer. DR: Diabetic retinopathy. DPN: Diabetic peripheral neuropathy.

Supplementary Table S4. Utilities used in analysis

| Utilities | Values | Ranges | References |
| --- | --- | --- | --- |
| Type 2 diabetes | 0.785 | 0.736-1 | (10) |
| Non type 2 diabetes | 1 | - | - |
| CVD | 0.73 | 0.639-0.822 | (11) |
| DKD | 0.78 | 0.624-0.936 | (10) |
| ESRD | 0.60 | 0.39-0.81 | (3) |
| DFU | 0.70 | 0.56-0.84 | (4) |
| Amputation | 0.62 | 0.5-0.74 | (4) |
| DR | 0.81 | 0.648-0.972 | (10) |
| Blindness | 0.26 | 0.208-0.312 | (12) |
| Stroke | 0.621 | 0.563-0.68 | (11) |
| Multiple complications | 0.59 | 0.57-0.61 | (13) |
| DPN | 0.74 | 0.592-0.888 | (12) |

CVD : Cardiovascular Disease. ESRD: End-Stage Renal Disease. DKD: Diabetic kidney disease. DFU: Diabetic foot ulcer. DR: Diabetic retinopathy. DPN: Diabetic peripheral neuropathy.

Supplementary Table S5. Transition probabilities of type 2 diabetes and complications in urban and rural area of China

| Transition probabilities | Urban | | Rural | | References |
| --- | --- | --- | --- | --- | --- |
|  | Values | Ranges | Values | Ranges |  |
| Type 2 diabetes mortality - Male | 0.00013 | 0.0001064-0.0001596 | 0.000088 | 0.0000704-0.0001056 | (14) |
| Type 2 diabetes mortality – Female | 0.00012 | 0.0000976-0.0001464 | 0.0001 | 0.00008-0.00012 | (14) |
| DFU | 0.081 | 0.056-0.106 | 0.1150 | 0.09202-0.13802 | (15) |
| Missed diagnosis DFU | 0.11267 | 0.09014-0.13521 | 0.15999 | 0.09014-0.13521 | (15) |
| DFU mortality | 0.1440 | 0.099-0.189 | 0.1440 | 0.099-0.189 | (15) |
| Missed diagnosis DFU mortality | 0.1858 | 0.14861-0.22291 | 0.1858 | 0.14861-0.22291 | (15) |
| Amputation | 0.051 | 0.02-0.082 | 0.051 | 0.02-0.082 | (15) |
| Missed diagnosis amputation | 0.0709 | 0.02782-0.11406 | 0.0709 | 0.02782-0.11406 | (15) |
| Amputation mortality - Male | 0.1850 | 0.148-0.222 | 0.1850 | 0.148-0.222 | (16) |
| Amputation mortality - Female | 0.2130 | 0.1704-0.2556 | 0.2130 | 0.1704-0.2556 |  |
| CVD 20-44y | 0.0055 | 0.0044-0.0066 | 0.00650 | 0.00503-0.007802 | (17) |
| CVD 45-64y | 0.0200 | 0.01598-0.02398 | 0.02358 | 0.018861-0.028292 |  |
| CVD 65-74y | 0.0217 | 0.0174-0.0261 | 0.0257 | 0.02049-0.03086 |  |
| CVD ≥75y | 0.0281 | 0.0225-0.0337 | 0.03317 | 0.02654-0.0398 |  |
| CVD mortality | 0.0790 | 0.0632-0.0948 | 0.0790 | 0.0632-0.0948 | (18) |
| Missed diagnosis CVD mortality | 0.1019 | 0.0815-0.1223 | 0.1019 | 0.0815-0.1223 | (18) |
| DKD-Male | 0.0204 | 0.0163-0.0245 | 0.0204 | 0.0163-0.0245 | (19) |
| DKD-Female | 0.0246 | 0.0197-0.0295 | 0.0246 | 0.0197-0.0295 |  |
| DKD mortality | 0.0503 | 0.0402-0.0604 | 0.0503 | 0.0402-0.0604 | (20) |
| Missed diagnosis DKD mortality | 0.0826 | 0.06605-0.09907 | 0.0826 | 0.06605-0.09907 | (20) |
| Stroke 20-44y | 0.00503 | 0.004024-0.006036 | 0.00629 | 0.00503-0.007545 | (17) |
| Stroke 45-64y | 0.00997 | 0.007976-0.011964 | 0.01246 | 0.00997-0.014955 |  |
| Stroke 65-74y | 0.01575 | 0.0126-0.0189 | 0.01969 | 0.01575-0.023625 |  |
| Stroke≥75y | 0.0289 | 0.02312-0.03468 | 0.0361 | 0.0289-0.04335 |  |
| Stroke mortality | 0.0325 | 0.026-0.039 | 0.0325 | 0.026-0.039 | (21) |
| Missed diagnosis stoke mortality | 0.04193 | 0.031347-0.052374 | 0.0419 | 0.031347-0.052374 | (21) |
| Blindness | 0.0200 | 0.002-0.03 | 0.0418 | 0.03135-0.05225 | (6) |
| Missed diagnosis blindness | 0.0278 | 0.022256-0.033384 | 0.05814 | 0.046515-0.06977 | (6) |
| DR | 0.0220 | 0.0176-0.0264 | 0.0220 | 0.0176-0.0264 | (22) |
| Missed diagnosis DR | 0.0306 | 0.02448-0.0367224 | 0.0306 | 0.02448-0.0367224 | (22) |
| DR mortality | 0.052 | 0.0416-0.0624 | 0.052 | 0.0416-0.0624 | (23) |
| ESRD | 0.00077 | 0.000616-0.000924 | 0.000886 | 0.000708-0.00106 | (24) |
| Missed diagnosis ESRD | 0.00107 | 0.0008346-0.001391 | 0.0012317 | 0.0009598-0.0016 | (24) |
| ESRD mortality | 0.2200 | 0.176-0.264 | 0.2200 | 0.176-0.264 | (25) |
| Missed diagnosis ESRD mortality | 0.2838 | 0.21285-0.35475 | 0.2838 | 0.21285-0.35475 | (25) |
| Multiple complications | 0.1425 | 0.114-0.171 | 0.1425 | 0.114-0.171 | (26) |
| Missed diagnosis multiple complications | 0.1982 | 0.15857-0.237861 | 0.19822 | 0.15857-0.237861 | (26) |
| Multiple complications mortality | 0.0785 | 0.0734-0.0839 | 0.0785 | 0.0734-0.0839 | (27) |
| Missed diagnosis multiple complications mortality | 0.1013 | 0.094686-0.108231 | 0.1013 | 0.094686-0.108231 | (27) |
| DPN | 0.0199 | 0.01592-0.02388 | 0.0199 | 0.01592-0.02388 | (12) |
| Missed diagnosis DPN | 0.0277 | 0.02076-0.034601 | 0.02768 | 0.02076-0.034601 | (12) |
| DPN mortality | 0.0200 | 0.016-0.024 | 0.0200 | 0.016-0.024 | (12) |
| Missed diagnosis DPN mortality | 0.0258 | 0.01935-0.03225 | 0.0258 | 0.01935-0.03225 | (12) |

CVD : Cardiovascular Disease. ESRD: End-Stage Renal Disease. DKD: Diabetic kidney disease. DFU: Diabetic foot ulcer. DR: Diabetic retinopathy. DPN: Diabetic peripheral neuropathy.

Supplementary Table S6A. Parameters for fitting β distributions in probabilistic sensitivity analysis

| Parameters | Value | α | β |
| --- | --- | --- | --- |
| FCG sensitivity | 65.1% | 6010 | 3222 |
| FCG specificity | 72.4% | 6684 | 2548 |
| POCT HbA1c sensitivity | 94.7% | 797 | 45 |
| POCT HbA1c specificity | 81.1% | 683 | 159 |
| Venous blood HbA1c sensitivity | 75.6% | 4544 | 1996 |
| Venous blood HbA1c specificity | 91.9% | 6010 | 530 |
| DFU transition probabilities | 8.1% | 36 | 409 |
| Amputation transition probabilities | 5.1% | 23 | 422 |
| DKD (Male) transition probabilities | 2.04% | 142 | 6822 |
| DKD (Female) transition probabilities | 2.46% | 171 | 6793 |

FCG: fasting capillary blood glucose. POCT: point-of-care test. DKD: Diabetic kidney disease. DFU: Diabetic foot ulcer.

Supplementary Table S6B. Parameters fitting the lognormal distribution (1)

| Cost | Value（$） | μ | σ |
| --- | --- | --- | --- |
| DKD | 1692 | 7.43 | 0.743 |
| ESRD | 14241 | 9.56 | 0.956 |
| DFU | 2334.7 | 7.76 | 0.776 |
| Amputation | 4307.4 | 8.37 | 0.837 |
| DR | 741.94 | 6.61 | 0.661 |
| Blindness | 3765 | 8.23 | 0.823 |
| CVD | 1175.65 | 7.07 | 0.707 |
| Multiple complications | 2994 | 8 | 0.8 |
| DPN | 1102 | 7.005 | 0.7005 |
| Stroke | 1995.47 | 7.6 | 0.76 |

CVD : Cardiovascular Disease. ESRD: End-Stage Renal Disease. DKD: Diabetic kidney disease. DFU: Diabetic foot ulcer. DR: Diabetic retinopathy. DPN: Diabetic peripheral neuropathy.

Supplementary Table S6B. Parameters fitting the lognormal distribution (2)

|  | Urban | | | Rural | | | |
| --- | --- | --- | --- | --- | --- | --- | --- |
| Cost | Value（$） | μ | σ | Value（$） | μ | | σ |
| Venous blood HbA1c | 59.98 | 4.09 | 0.409 | 82.01 | 4.41 | 0.441 | |
| Venous blood HbA1c+OGTT | 113.3 | 4.73 | 0.473 | 159.40 | 5.07 | | 0.507 |
| POCT HbA1c | 4.34 | 1.47 | 0.147 | 4.34 | 1.47 | | 0.147 |
| POCT HbA1c+OGTT | 61.1 | 4.11 | 0.411 | 83.13 | 4.42 | | 0.442 |
| FCG | 3.24 | 1.18 | 0.118 | 3.24 | 1.18 | | 0.118 |
| FCG+OGTT | 60 | 4.09 | 0.409 | 82.03 | 4.41 | | 0.441 |
| Diabetes- Venous blood HbA1c+OGTT | 477.3 | 6.17 | 0.617 | 519.96 | 6.25 | | 0.625 |
| Diabetes -POCT HbA1c+OGTT | 425.1 | 6.05 | 0.605 | 447.13 | 6.10 | | 0.610 |
| Diabetes - FCG+OGTT | 424 | 6.05 | 0.605 | 446.03 | 6.10 | | 0.610 |

FCG: fasting capillary blood glucose. POCT: point-of-care test. OGTT: oral glucose tolerance test.

Supplementary Table S6C. Parameters fitting uniform distribution - Transition probabilities

| Parameters | Urban | | Rural | |
| --- | --- | --- | --- | --- |
|  | Value | Ranges | Value | Ranges |
| Missed diagnosis amputation | 0.070941 | 0.02782-0.11406 | 0.070941 | 0.02782-0.11406 |
| Amputation mortality - Male | 0.185 | 0.148-0.222 | 0.185 | 0.148-0.222 |
| Amputation mortality - Female | 0.213 | 0.1704-0.2556 | 0.213 | 0.1704-0.2556 |
| CVD 20-44y | 0.00551 | 0.0044-0.0066 | 0.0065018 | 0.00503-0.007802 |
| CVD 45-64y | 0.01998 | 0.01598-0.02398 | 0.0235764 | 0.018861-0.028292 |
| CVD 65-74y | 0.02171 | 0.0174-0.0261 | 0.025718 | 0.02049-0.03086 |
| CVD ≥75y | 0.02811 | 0.0225-0.0337 | 0.0331698 | 0.02654-0.0398 |
| CVD mortality | 0.079 | 0.0632-0.0948 | 0.079 | 0.0632-0.0948 |
| Missed diagnosis CVD mortality | 0.12513 | 0.100104-0.15016 | 0.12513 | 0.100104-0.15016 |
| DKD mortality | 0.064 | 0.062-0.0683 | 0.064 | 0.062-0.0683 |
| Missed diagnosis DKD mortality | 0.08256 | 0.06605-0.09907 | 0.08256 | 0.06605-0.09907 |
| Type 2 diabetes prevalence rate | 0.137 | 0.1096-0.1644 | 0.12 | 0.096-0.144 |
| Type 2 diabetes mortality - Male | 0.000133 | 0.0001064-0.0001596 | 0.000088 | 0.0000704-0.0001056 |
| Type 2 diabetes mortality – Female | 0.000122 | 0.0000976-0.0001464 | 0.0001 | 0.00008-0.00012 |
| Stroke 20-44y | 0.00503 | 0.004024-0.006036 | 0.0062876 | 0.00503-0.007545 |
| Stroke 45-64y | 0.00997 | 0.007976-0.011964 | 0.0124625 | 0.00997-0.014955 |
| Stroke 65-74y | 0.01575 | 0.0126-0.0189 | 0.0196875 | 0.01575-0.023625 |
| Stroke≥75y | 0.0289 | 0.02312-0.03468 | 0.036125 | 0.0289-0.04335 |
| Stroke mortality | 0.0325 | 0.026-0.039 | 0.0325 | 0.026-0.039 |
| Missed diagnosis stoke mortality | 0.041925 | 0.031347-0.052374 | 0.041925 | 0.031347-0.052374 |
| Blindness | 0.02 | 0.002-0.03 | 0.0418 | 0.03135-0.05225 |
| Missed diagnosis blindness | 0.02782 | 0.022256-0.33384 | 0.0581438 | 0.046515-0.06977 |
| DR | 0.022 | 0.0176-0.0264 | 0.022 | 0.0176-0.0264 |
| Missed diagnosis DR | 0.030602 | 0.02448-0.0367224 | 0.030602 | 0.02448-0.0367224 |
| DR mortality | 0.052 | 0.0416-0.0624 | 0.052 | 0.0416-0.0624 |
| ESRD | 0.00077 | 0.000616-0.000924 | 0.0008855 | 0.000708-0.00106 |
| Missed diagnosis ESRD | 0.001071 | 0.0008346-0.001391 | 0.00123173 | 0.0009598-0.0016 |
| ESRD mortality | 0.22 | 0.176-0.264 | 0.22 | 0.176-0.264 |
| Missed diagnosis ESRD mortality | 0.2838 | 0.21285-0.35475 | 0.2838 | 0.21285-0.35475 |
| Multiple complications | 0.1425 | 0.114-0.171 | 0.1425 | 0.114-0.171 |
| Missed diagnosis multiple complications | 0.198218 | 0.15857-0.237861 | 0.198218 | 0.15857-0.237861 |
| Multiple complications mortality | 0.0785 | 0.0734-0.0839 | 0.0785 | 0.0734-0.0839 |
| Missed diagnosis multiple complications mortality | 0.101265 | 0.094686-0.108231 | 0.101265 | 0.094686-0.108231 |
| DPN | 0.0199 | 0.01592-0.02388 | 0.0199 | 0.01592-0.02388 |
| Missed diagnosis DPN | 0.027681 | 0.02076-0.034601 | 0.027681 | 0.02076-0.034601 |
| DPN mortality | 0.02 | 0.016-0.024 | 0.02 | 0.016-0.024 |
| Missed diagnosis DPN mortality | 0.0258 | 0.01935-0.03225 | 0.0258 | 0.01935-0.03225 |

CVD : Cardiovascular Disease. ESRD: End-Stage Renal Disease. DKD: Diabetic kidney disease. DFU: Diabetic foot ulcer. DR: Diabetic retinopathy. DPN: Diabetic peripheral neuropathy.

Supplementary Table S6C. Parameters fitting uniform distribution – Utilities

| Parameters | Value | Ranges |
| --- | --- | --- |
| Type 2 diabetes | 0.785 | 0.736-1 |
| CVD | 0.73 | 0.639-0.822 |
| DKD | 0.78 | 0.624-0.936 |
| ESRD | 0.60 | 0.39-0.81 |
| DFU | 0.70 | 0.56-0.84 |
| Amputation | 0.62 | 0.5-0.74 |
| DR | 0.81 | 0.648-0.972 |
| Blindness | 0.26 | 0.208-0.312 |
| Stroke | 0.621 | 0.563-0.68 |
| Multiple complications | 0.59 | 0.57-0.61 |
| DPN | 0.74 | 0.592-0.888 |

CVD : Cardiovascular Disease. ESRD: End-Stage Renal Disease. DKD: Diabetic kidney disease. DFU: Diabetic foot ulcer. DR: Diabetic retinopathy. DPN: Diabetic peripheral neuropathy.


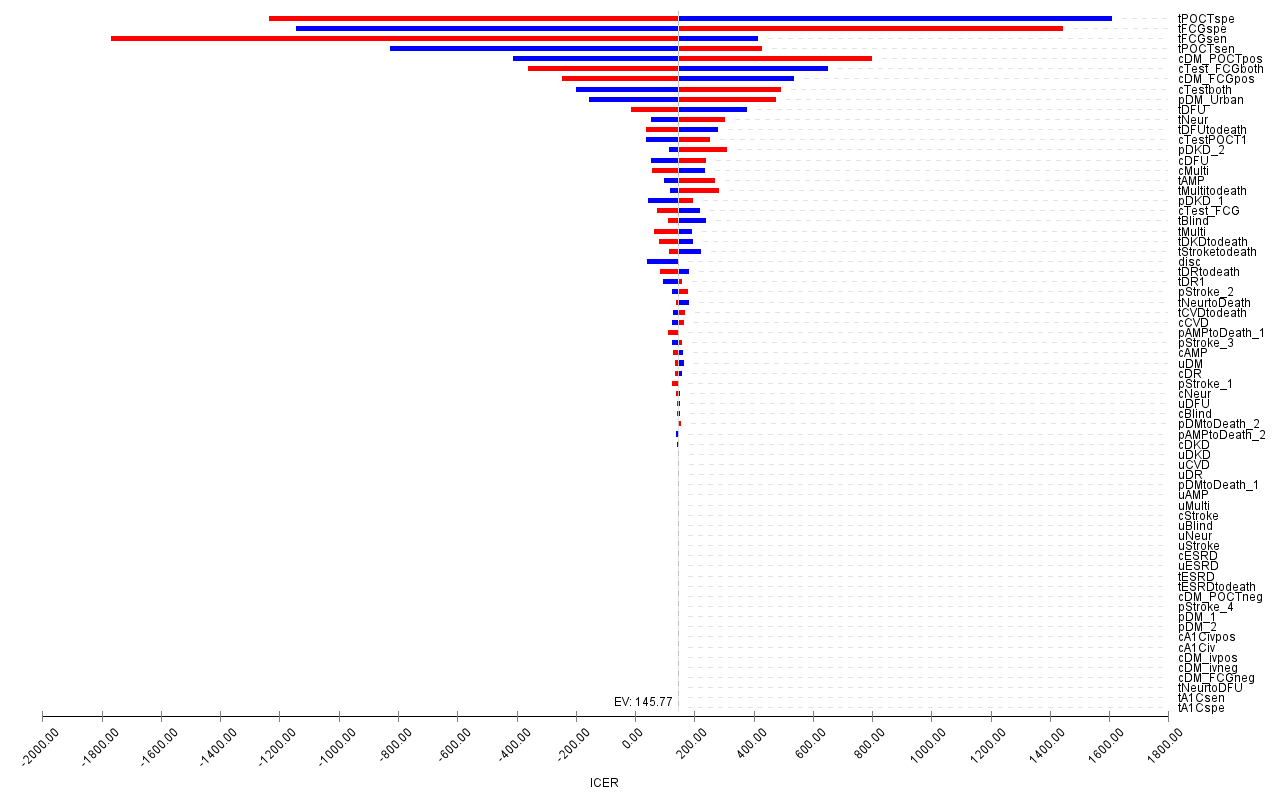


**Supplementary Figure 1a. Tornado diagrams of FCG vs. POCT HbA1c one-way sensitivity analysis in urban area.** tPOCTspe: Specificity of POCT HbA1c in type 2 diabetes diagnosis. tFCGspe: Specificity of FCG in type 2 diabetes diagnosis. tFCGsen: Sensitivity of FCG in type 2 diabetes diagnosis. tPOCTsen: Sensitivity of POCT HbA1c in type 2 diabetes diagnosis. cDM_POCTpos : Cost of diagnosing type 2 diabetes in POCT HbA1c-positive individuals.


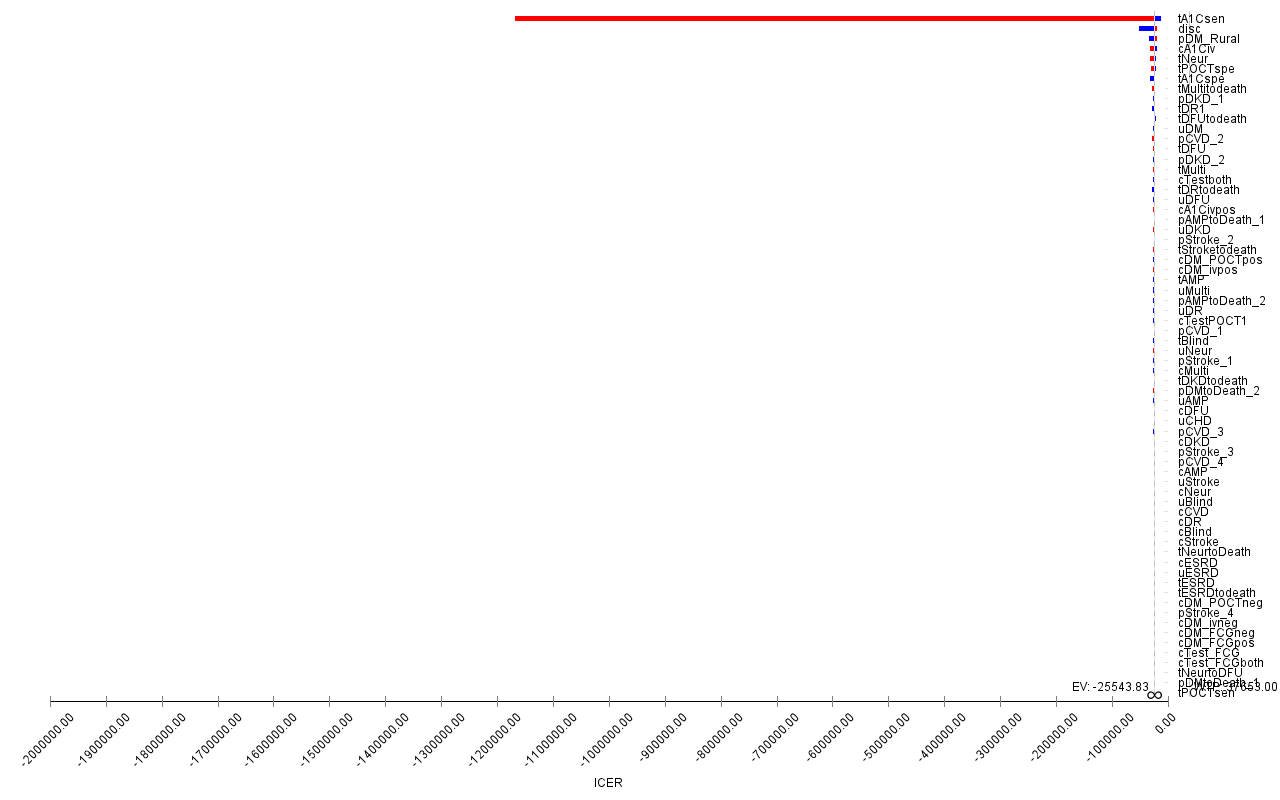


**Supplementary Figure 1b. Tornado diagrams of venous blood HbA1c vs. POCT HbA1c one-way sensitivity analysis in urban area.** tA1Csen: Sensitivity of venous blood HbA1c in type 2 diabetes diagnosis. disc : discount rate. pDM_rural : prevalence of type 2 diabetes in rural areas. cA1Civ : Cost of venous blood HbA1c testing. tNeur: transition probability of diabetic peripheral neuropathy.


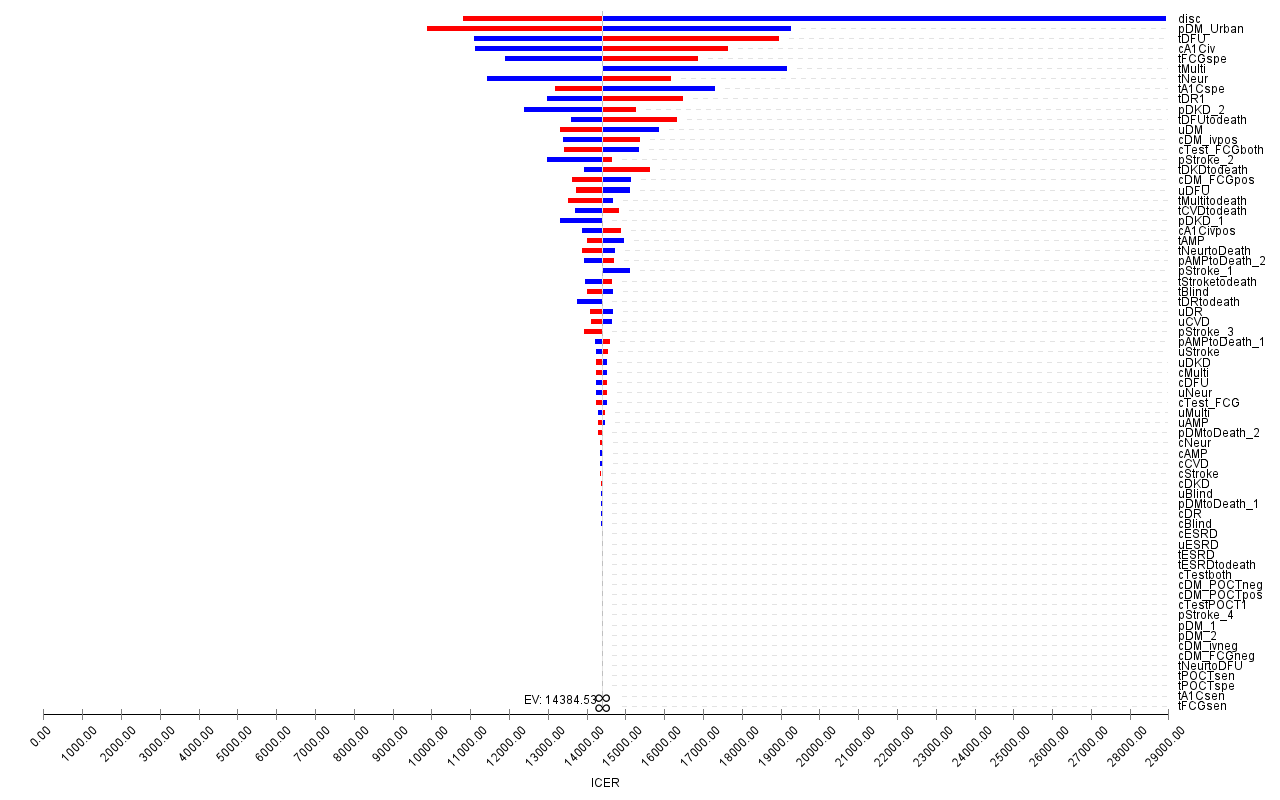


**Supplementary Figure 1c. Tornado diagrams of venous blood HbA1c vs. FCG one-way sensitivity analysis in urban area.** disc : discount rate. pDM_Urban : prevalence of type 2 diabetes in urban areas. tDFU: transition probability of diabetic foot ulcer. cA1Civ : Cost of venous blood HbA1c testing. tFCGspe: Specificity of FCG in type 2 diabetes diagnosis.

**
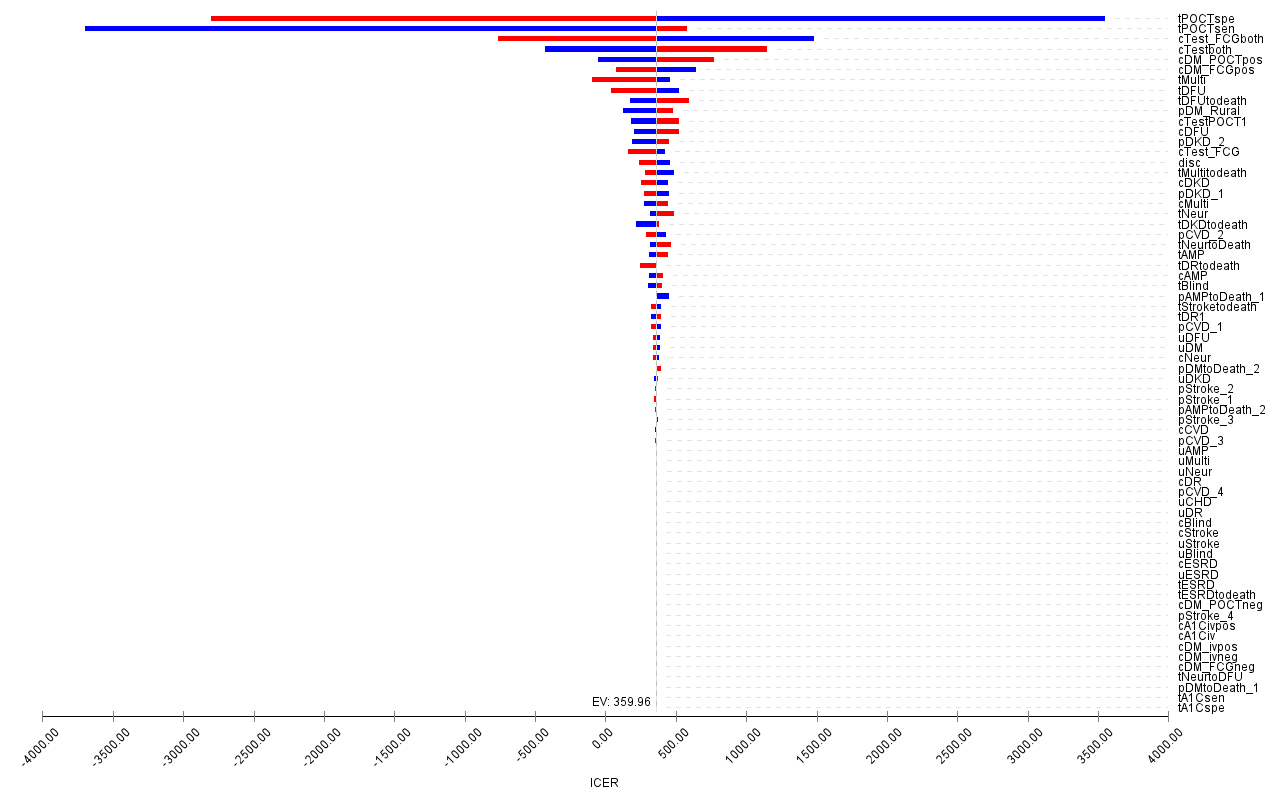
**

**Supplementary Figure 1d. Tornado diagrams of FCG vs. POCT HbA1c one-way sensitivity analysis in rural area.** tPOCTspe: Specificity of POCT HbA1c in type 2 diabetes diagnosis. tPOCTsen: Sensitivity of POCT HbA1c in type 2 diabetes diagnosis. cTest_FCGboth : Cost of both FCG and OGTT testing. cDM_POCTpos : Cost of diagnosing type 2 diabetes in POCT HbA1c-positive individuals. cDM_FCGpos : Cost of diagnosing type 2 diabetes in FCG-positive individuals.

**
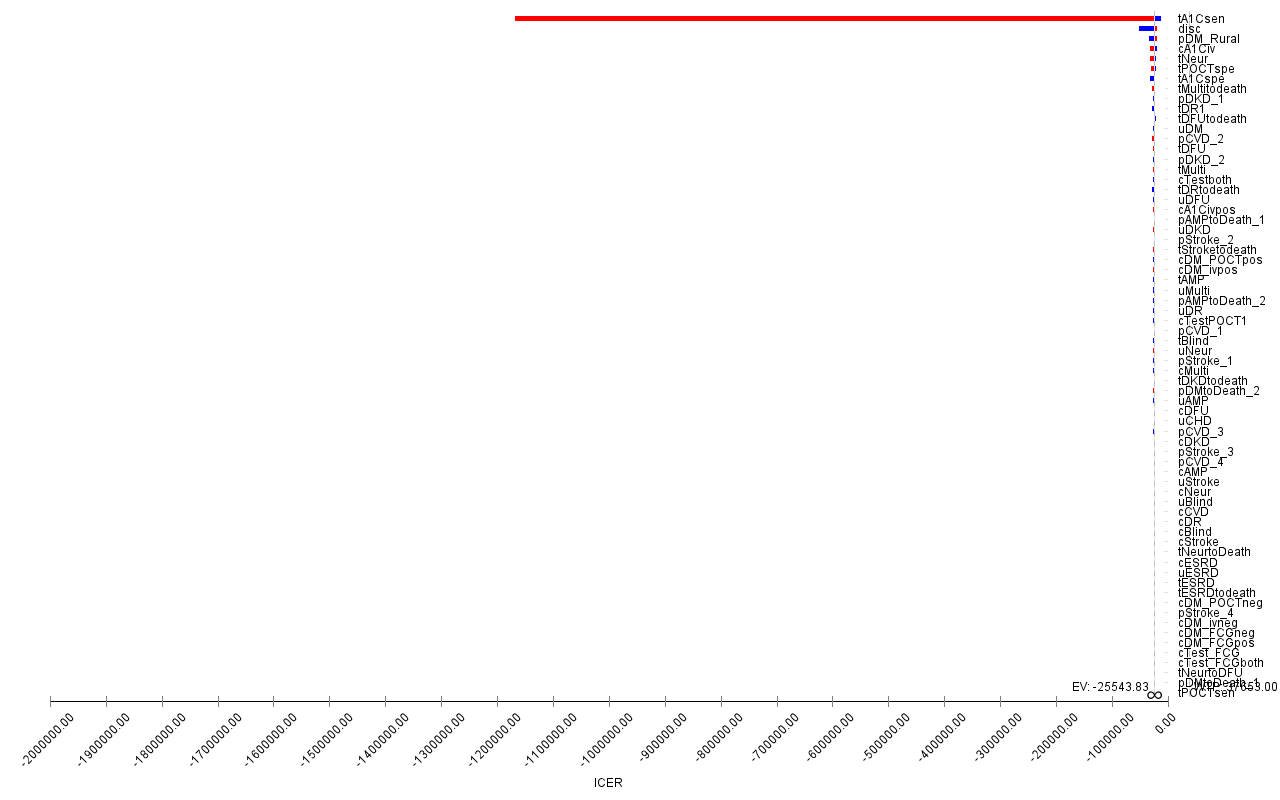
**

**Supplementary Figure 1e. Tornado diagrams of venous blood HbA1c vs. POCT HbA1c one-way sensitivity analysis in rural area.** tA1Csen: Sensitivity of venous blood HbA1c in type 2 diabetes diagnosis. disc : discount rate. pDM_rural : prevalence of type 2 diabetes in rural areas. cA1Civ : Cost of venous blood HbA1c testing. tNeur: transition probability of diabetic peripheral neuropathy.

**
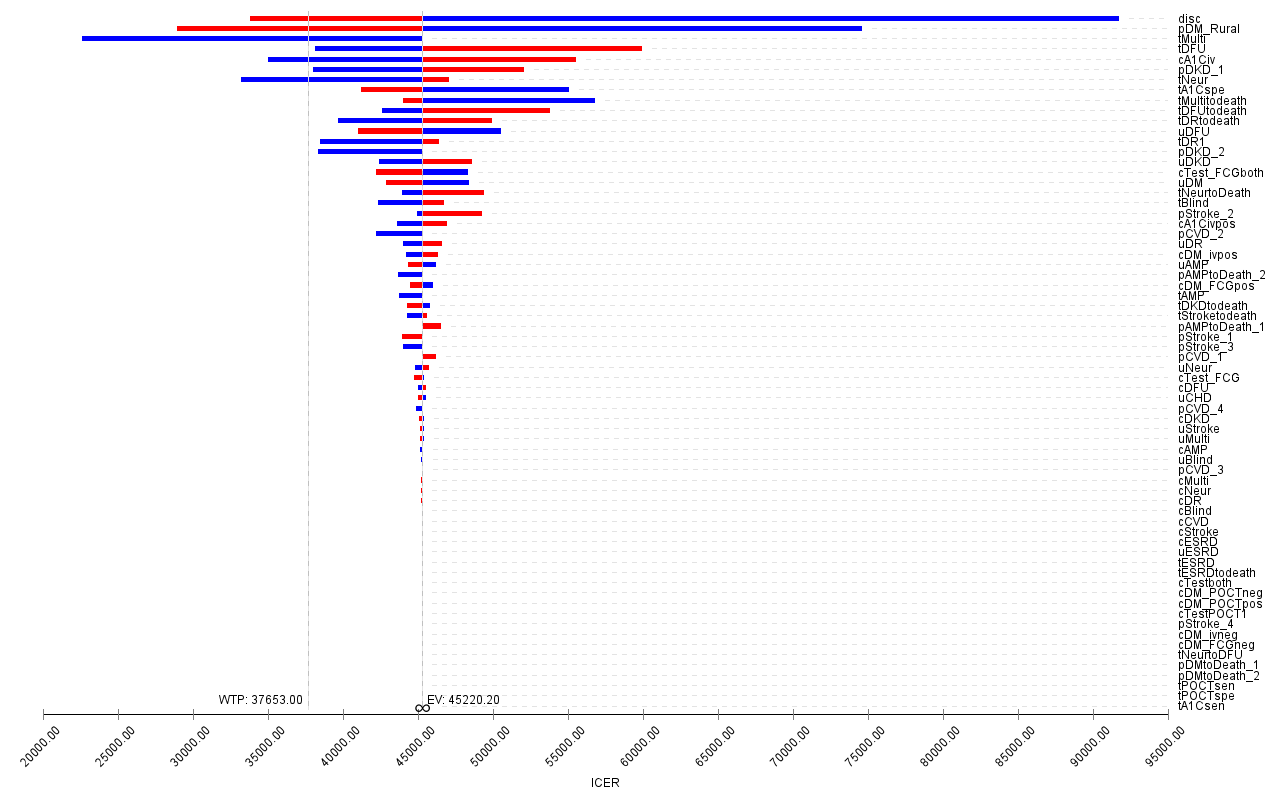
**

**Supplementary Figure 1f. Tornado diagrams of venous blood HbA1c vs. FCG one-way sensitivity analysis in rural area.** disc : discount rate. pDM_Urban : prevalence of type 2 diabetes in urban areas. tMulti: transition probability of multiple complications. tDFU: transition probability of diabetic foot ulcer. cA1Civ : Cost of venous blood HbA1c testing.

**References**

1. Guo L, Zheng J, Pan Q, Zhang Q, Zhou Y, Wang W, et al. Changes in Direct Medical Cost and Medications for Managing Diabetes in Beijing, China, 2016 to 2018: Electronic Insurance Data Analysis. Ann Fam Med. 2021;19(4):332-41.

2. Wu H, Eggleston KN, Zhong J, Hu R, Wang C, Xie K, et al. How do type 2 diabetes mellitus (T2DM)-related complications and socioeconomic factors impact direct medical costs? A cross-sectional study in rural Southeast China. BMJ Open. 2018;8(11):e020647.

3. Wu B, Zhang S, Lin H, Mou S. Prevention of renal failure in Chinese patients with newly diagnosed type 2 diabetes: A cost-effectiveness analysis. Journal of Diabetes Investigation. 2018;9(1):152-61.

4. Wu B, Wan X, Ma J. Cost-effectiveness of prevention and management of diabetic foot ulcer and amputation in a health resource-limited setting. J Diabetes. 2018;10(4):320-7.

5. Wang A-h, Xu Z-r, Ji L-n. [Clinical characteristics and medical costs of diabetics with amputation at central urban hospitals in China]. Zhonghua Yi Xue Za Zhi. 2012;92(4):224-7.

6. Wu B, Li J, Wu H. Strategies to Screen for Diabetic Retinopathy in Chinese Patients with Newly Diagnosed Type 2 Diabetes: A Cost-Effectiveness Analysis. Medicine (Baltimore). 2015;94(45):e1989.

7. Huang X-M, Yang B-F, Zheng W-L, Liu Q, Xiao F, Ouyang P-W, et al. Cost-effectiveness of artificial intelligence screening for diabetic retinopathy in rural China. BMC Health Serv Res. 2022;22(1):260.

8. Qin J, Zhang Y, Zhang L, Rui D, Mao L, Wang L, et al. Analysis of the Prevalence of Chronic Diseases in Typical Urban Residents and the Burden of Disease on Patients. Chinese Journal of Public Health. 2014;30(01):5-7.

9. Zhu D, Shi X, Nicholas S, Chen S, Ding R, Huang L, et al. Medical Service Utilization and Direct Medical Cost of Stroke in Urban China. Int J Health Policy Manag. 2022;11(3):277-86.

10. Zhang Y, Wu J, Chen Y, Shi L. EQ-5D-3L Decrements by Diabetes Complications and Comorbidities in China. Diabetes Ther. 2020;11(4):939-50.

11. Wu B, Shi L. Cost-utility of ticagrelor plus aspirin in diabetic patients with stable coronary artery disease. Eur Heart J Cardiovasc Pharmacother. 2021;7(6):529-38.

12. Rodríguez-Sánchez B, Peña-Longobardo LM, Sinclair AJ. Cost-effectiveness analysis of the Neuropad device as a screening tool for early diabetic peripheral neuropathy. Eur J Health Econ. 2020;21(3):335-49.

13. Koopmanschap M. Coping with Type II diabetes: the patient's perspective. Diabetologia. 2002;45(Suppl 1):S21-S2.

14. Su B, Wang Y, Dong Y, Hu G, Xu Y, Peng X, et al. Trends in Diabetes Mortality in Urban and Rural China, 1987-2019: A Joinpoint Regression Analysis. Front Endocrinol (Lausanne). 2021;12:777654.

15. Jiang Y, Wang X, Xia L, Fu X, Xu Z, Ran X, et al. A cohort study of diabetic patients and diabetic foot ulceration patients in China. Wound Repair Regen. 2015;23(2):222-30.

16. Wu H, Yang A, Lau ESH, Ma RCW, Kong APS, Chow E, et al. Secular trends in rates of hospitalisation for lower extremity amputation and 1 year mortality in people with diabetes in Hong Kong, 2001-2016: a retrospective cohort study. Diabetologia. 2020;63(12):2689-98.

17. Wu H, Lau ESH, Yang A, Ma RCW, Kong APS, Chow E, et al. Trends in diabetes-related complications in Hong Kong, 2001-2016: a retrospective cohort study. Cardiovasc Diabetol. 2020;19(1):60.

18. McMurray JJV, Solomon SD, Inzucchi SE, Køber L, Kosiborod MN, Martinez FA, et al. Dapagliflozin in Patients with Heart Failure and Reduced Ejection Fraction. N Engl J Med. 2019;381(21):1995-2008.

19. Lin C-C, Chen C-C, Chen F-N, Li C-I, Liu C-S, Lin W-Y, et al. Risks of diabetic nephropathy with variation in hemoglobin A1c and fasting plasma glucose. Am J Med. 2013;126(11):1017.e1-.10.

20. González-Pérez A, Saez M, Vizcaya D, Lind M, Garcia Rodriguez L. Incidence and risk factors for mortality and end-stage renal disease in people with type 2 diabetes and diabetic kidney disease: a population-based cohort study in the UK. BMJ Open Diabetes Res Care. 2021;9(1).

21. Di Angelantonio E, Kaptoge S, Wormser D, Willeit P, Butterworth AS, Bansal N, et al. Association of Cardiometabolic Multimorbidity With Mortality. JAMA. 2015;314(1):52-60.

22. Xu J, Xu L, Wang YX, You QS, Jonas JB, Wei WB. Ten-year cumulative incidence of diabetic retinopathy. The Beijing Eye Study 2001/2011. PLoS One. 2014;9(10):e111320.

23. Sabanayagam C, Chee ML, Banu R, Cheng C-Y, Lim SC, Tai ES, et al. Association of Diabetic Retinopathy and Diabetic Kidney Disease With All-Cause and Cardiovascular Mortality in a Multiethnic Asian Population. JAMA Netw Open. 2019;2(3):e191540.

24. Cheng H-T, Xu X, Lim PS, Hung K-Y. Worldwide Epidemiology of Diabetes-Related End-Stage Renal Disease, 2000-2015. Diabetes Care. 2021;44(1):89-97.

25. Liu Y, Wang L, Han X, Wang Y, Sun X, Jiang H, et al. The Profile of Timing Dialysis Initiation in Patients with End-stage Renal Disease in China: A Cohort Study. Kidney Blood Press Res. 2020;45(2):180-93.

26. Luijks H, Schermer T, Bor H, van Weel C, Lagro-Janssen T, Biermans M, et al. Prevalence and incidence density rates of chronic comorbidity in type 2 diabetes patients: an exploratory cohort study. BMC Med. 2012;10:128.

27. Coles B, Zaccardi F, Hvid C, Davies MJ, Khunti K. Cardiovascular events and mortality in people with type 2 diabetes and multimorbidity: A real-world study of patients followed for up to 19 years. Diabetes Obes Metab. 2021;23(1):218-27.
